# International R&D Collaboration for a Global Aging Society: Focusing on Aging-Related National-Funded Projects

**DOI:** 10.3390/ijerph17228545

**Published:** 2020-11-18

**Authors:** Doyeon Lee, Seungwook Kim, Keunhwan Kim

**Affiliations:** 1 Division of Data Analysis, Korea Institute of Science and Technology Information (KISTI), Seoul 02456, Korea; dylee@kisti.re.kr; 2 Department of Datamining, Rloha LLC., Seoul 08760, Korea; ceo@rloha.io

**Keywords:** aging society, national-funded project data, international collaboration, interdisciplinary, cluster analysis, usable information

## Abstract

An international research and development (R&D) collaboration for aging-related projects is necessary to alleviate the severe economic/healthcare/humanitarian challenges of a global aging society. This study presents a practical/systematic framework that enables the provision of information on the research goals, the status of science and technology, and action plans of aging-related program development processes. We used data on aging-related national-funded projects from the United States of America, the European Union, the United Kingdom, Japan, and Korea. We collected data on 6318 national-funded projects, subsequently designating research fields to each project. By analyzing the content of the projects, their representative research fields, and the associated keywords, we assessed the general goals of six different research fields. To recognize the current scientific capabilities of these research fields, we divided the projects by clusters. We provided information on research organizations, specific goals (i.e., project title), project periods, and the funding related to the projects. These may be used by stakeholders in various governments/institutions/industries during future discussions regarding the establishment of an international R&D collaboration strategy. The approach we proposed may facilitate the linkage between knowledge and action during strategy development by maximizing scientific legitimacy, developing consensual knowledge, and minimizing diverging opinions among stakeholders.

## 1. Introduction

Accelerated economic development and advances in healthcare, as well as breakthroughs in the scientific and medicine fields, have allowed for people in the modern world to live longevous lives [1]. As global life expectancy increased, the number of older adults diagnosed with neurodegenerative diseases also dramatically surged [2]. These diseases stem from the progressive degeneration and/or death of nerve cells over time, thereby jeopardizing, partially or entirely, the normal functioning of the brain; this may, in turn, generate the onset of dementia. Some examples of dementia include Alzheimer’s, Parkinson’s, and Huntington’s disease [3]. Given such clinical characteristics and the increase in the population of older people worldwide, it is plausible to infer that this situation brings negative consequences, which thereby aggravate the societal burden regarding healthcare management, improvements in the social support system, and in the expansion of public health infrastructure [1,4,5].

Studies have shown that owing to the increase in numbers of the population of people living with dementia worldwide—whom negatively impact their families and societies—many nations are being compelled to cope with this societal challenge [6,7]. Showing the gravity of the problem, a study showed that the estimated global cost of dementia is expected to reach 2 trillion United States Dollars (USD) by 2030 [6]. Based on the classification set forth by the United Nations, the societal costs of dementia in the United States of America (USA) were estimated to range from 157–215 billion USD in 2010, and these costs were estimated to revolve around 177 billion Euros across all of the European Union (EU) in 2008 [8]. Additionally, the Alzheimer’s Association remarked that the estimated healthcare cost—including long-term and hospice care—for Alzheimer’s and other dementias in the USA revolved around 290 billion USD in 2019 [9]. Owing to such high economic burden, many nations have been addressing population aging, and the challenges that it evokes, with utmost priority [1]. Accordingly, a study remarked the need to raise awareness about the significance of rigorous cross-national scientific research and policy dialogue to facilitate the dealing with the challenges that come with an aging world [10]. Fortunately, there is a generalized consensus that technology can meliorate the situation of countries that are currently confronting the challenges of population aging [1,11]. Thus, we believe that international research and development (R&D) activities should be established for the topic of aging-related diseases (e.g., degenerative aging and prevention of major aging-related diseases), as these may be necessary to alleviate the severe economic-, healthcare-, and humanitarian-related challenges that come with a global aging society. Consequently, encouraging R&D activities on this topic have become an urgent mission for nations worldwide. Thus, we now delineate some of the recent aging-related R&D efforts conducted by different countries and regions in the world.

In 2019, more than one-fifth (20.3%) of the population across all 27 member states of the EU was aged 65 and over, and this number is estimated to reach 24.2% by 2030 [12]; namely, the aforementioned burden of chronic diseases—which stems from an aging population—will be a future challenge for the healthcare systems of many European countries. Recently, these diseases were shown to already account for an estimated 70–80% of the healthcare cost in the EU [13]. Accordingly, in 2013, the EU launched the European Innovation Partnership on Active and Healthy Aging (EIP on AHA), which had the major goal of encouraging the broadening of partnerships and stakeholder engagements to deal with this societal challenge; it focused, particularly, on the promotion of healthy and active aging to foster older people’s continued economic contribution [14]. Together with the EIP on AHA, some other programs in the EU, such as the AAL (Active Assisted Living Programme) and the JPI MYBL (The Joint Programming Initiative (JPI) “More Years, Better Lives”), have contributed for a continued R&D agenda on active and healthy aging.

In 2019, 18.5% of the population in the United Kingdom (UK) was aged 65 and over, and this number is estimated to reach 21.5% by 2030 [15]. Accordingly, the UK has several research councils and health departments which have shown a long-standing commitment to funding aging-related R&D activities, such as the Biotechnology and Biological Sciences Research Council (BBSRC), the Engineering and Physical Sciences Research Council (EPSRC), and the Medical Research Council (MRC). The largest programs of research, such as the New Dynamics of Ageing (NDA), Lifelong Health and Wellbeing (LLHW), and Advanced Pain Discovery Platform (APDP) have provided to implement the political definition of active aging [16]. Moreover, the current government in the UK positioned aging as one of the four “grand challenges” that were to be addressed in 2019 [17].

In 2019, 16.2% of the American population was aged 65 and over, and this number is estimated to reach 20.3% by 2030 [15]. Accordingly, in 2011, there were many efforts to prioritize older adults’ health and well-being at the national-level in the USA, including programs such as the Older Americans Act, the USA Department of Health and Human Services (HHS)’s Healthy Aging Summit, and the Healthy Aging in Action. Despite the national-level attention that these programs brought to the importance of older adults’ health, they were developed and conducted by different agencies, so they remained siloed and non-collaborative. Additionally, regardless of the variety of programs on aging-related topics that are active in the country, most operate independently, such as the Age-Friendly Communities, Centers for Disease Control and Prevention’s Healthy Brain Initiative, Dementia-Friendly Communities, Age-Friendly Health Systems, among others [18].

In 2019, 28.0% of the Japanese population was aged 65 and over, and this number is estimated to reach 30.9% by 2030 [15]. Accordingly, the Japanese government launched Japan’s 5th science and technology basic plan, named Society 5.0, to overcome the social challenges caused by an aging society [19]. Additionally, the Japan Science and Technology Agency (JST) has been funding a variety of R&D programs to solve issues regarding Japan’s super-aged society [20], such as the Moonshot [21], S-innovation, and the Grant-in-Aid for Scientific Research (KAKENHI).

In 2019, 15.1% of the Korean population was aged 65 and over, and this number is estimated to reach 24.7% by 2030 [15]. Thus, to tackle the challenges that come with an aging society, the Korean government launched the 3rd five-year plan for an Ageing Society and Population (2006–2020) [22]. Additionally, in 2020, the Ministry of Science and Information & Communications Technology (ICT) announced the plan called “Science and Technology Future Strategies 2045,” which covered the eight major challenges that Korea will face in the coming future; one of such challenges relates to research on healthy aging and the five most prevalent types of cancer [23]. As another R&D measure, in 2018, the same Ministry released the “3rd Korea Brain Initiative,” which aimed to conduct basic studies on the underlying mechanisms of decision-making, clinical studies on neurodegenerative diseases (e.g., Alzheimer’s and Parkinson’s disease), and studies on the development of novel neural technologies that are to be applied in these basic and clinical studies [24].

Regardless of such national-level efforts, a study remarked that fostering research on aging and aging-related diseases requires the promotion of international knowledge and technology exchange and attracting international resources; nonetheless, these can be achieved only if nations have greater commitment to multidisciplinary collaboration across organizations, research fields, and countries [25]. Consequently, major organizations and/or nations have established strategies to implement their proposed action plans on aging and health with other—and sometimes international—bodies [11,26]. For example, to create a set of global priorities for aging-related issues and strengthen the cooperation between multidisciplinary partners (e.g., international, intergovernmental, and nongovernmental organizations), the WHO set up the “Global strategy and action plan on ageing and health” [25]. Additionally, one of the leading aging-related organizations in the world, the National Institute on Aging (NIA) in the USA, emphasized the need for multidisciplinary collaborative research—conducted by international groups—to improve our understanding of the aging brain, Alzheimer’s disease, aging-related dementias, and other neurodegenerative diseases [26].

Two other studies also remarked that, when policy-makers or R&D program developers choose or need to carry out a periodic review on the progress of the implementation of strategies/programs related to aging and aging-related diseases—or to even develop a new strategy/program (i.e., strategy planning) on the topic—it is crucial for them to acquire a wide range of scientific evidence, as this may ensure appropriate improvements in their decision-making regarding the directions of these strategies/programs [27,28]. On the topic, a study remarked that a strategy planning starts with the answering of three fundamental questions: (a) Where are we going? (i.e., what are our goals; in this study, we provide data in this topic by the process of labeling) (b) Where are we now? (i.e., the present state of science and technology, or the interdisciplinarity of sciences; in this study, we provide information on this topic by using the ASJC (All Science Journal Classification Codes)) and (c) How can we get there? (i.e., what will be the coordinated action plans; in this study, we provide information on the title, abstract, funding, and periods of current R&D activities, also highlighting potential candidate partners) [29]. Another piece of research highlighted that strategic planning can be understood as a process that promotes policy coherence, secures the quality and legitimacy of public policies, and supports key policies by ensuring proper coordination between various organizations [30].

Based on prior research [28], to establish an R&D strategy planning, one needs to first identify the status quo in the targeted research fields; then, to reduce stakeholders’ uncertainty regarding the information on the different statuses of different targeted fields of knowledge, one needs to provide comprehensive evidence on the current status of the targeted research fields. As remarked by a prior study [31], this procedure is the cornerstone that ensures the strengthening of the coordination among different research fields, which thereby improves the quality of the decision-making process related to the R&D strategy planning [30]. Thus, we aimed to provide timely, comprehensive, and usable information about the status of R&D activities on aging-related diseases in five nations (i.e., the USA, EU, UK, Japan, and Korea) between the years of 2012–2019. Our research addresses the following questions:

(Goal) What are the particular goals of different research fields regarding aging and aging-related diseases from a global perspective?
(Status of science and technology) What scientific and technological fields have been utilized by different research fields to achieve these goals related to aging and aging-related diseases?
(Action plans) What are the research projects developed in different research fields that regard aging and aging-related diseases and that have been collaborative (i.e., regarding the exchange with other research organizations, their project periods, and funding)? What are the organizations in different research fields that may serve as multicounty and multidisciplinary partners for R&D collaboration on aging and aging-related diseases?

## 2. Materials and Methods

### 2.1. Data Collection and Preprocessing

We used data on national-funded R&D projects, and these were collected from the following R&D databases: The global R&D database provided by STAR METRICS (Science and Technology for America’s Reinvestment: Measuring the EffecTs of Research on Innovation, Competitiveness and Science); the NSF (National Science Foundation), which is a database from the USA; the CORDIS (Community Research and Development Information Service), which is a database from the EU; the Gateway to Research (GtR), which is a database from the UK; the KAKEN (Database of Grants-in-Aid for Scientific Research), which is a database from Japan; and the NTIS (National Science and Technology Information Service), which is a database from Korea.

**Figure 1:**
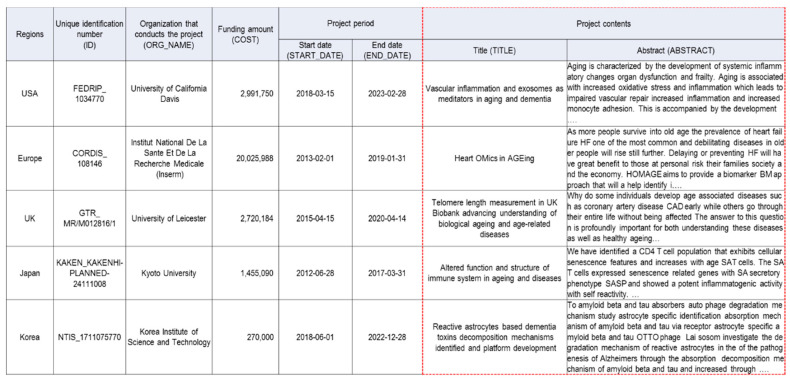
Examples of data on R&D public projects in the global R&D database.

The global R&D database was built and is operated internally by the KISTI (Korea Institute of Science and Technology Information), and it was funded by the Ministry of Science and ICT of Korea. It has data from approximately 1.8 million national-funded projects that went on between 2012 and 2019; the detailed process of database establishment is described in a prior study [32]. Examples of data on R&D public projects in the global R&D database are shown in Figure 1. In this study, we used the title and abstract of the R&D projects to assign them to specific research fields—which were represented by the 344 ASJC (All Science Journal Classification Codes) from Scopus.

In total, we collected information from 114,625 national-funded R&D projects related to aging and aging-related diseases that were conducted between 2012 and 2019 from the global R&D database. We used the search terms present in Table 1. After removing duplicated projects (i.e., with duplicated titles or organizations), projects with missing information on their funding, and small-sized projects (i.e., with a funding less than US $100,000), we acquired a final data sample of 6318 projects (Table 1).

**Figure 2:**
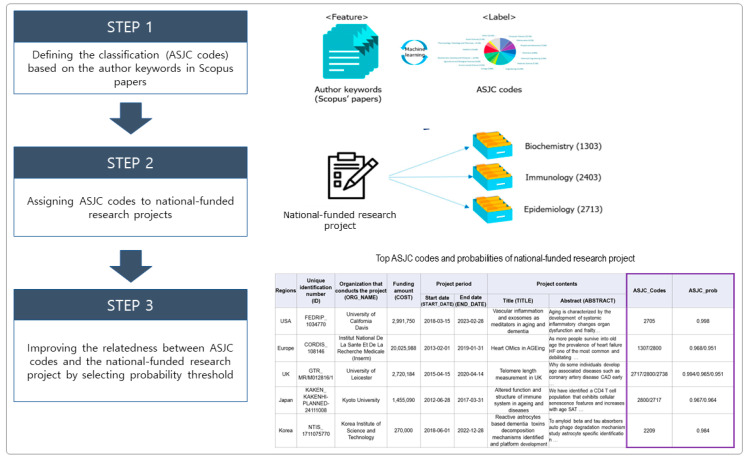
The process of assigning ASJC codes to national-funded research projects and improving the relatedness between the ASJC codes and the projects.

To understand the interdisciplinary characteristics of the research fields related to these national-funded research projects, we used the ASJC classification model in the machine learning process by employing the Author keywords from approximately one million articles found in Scopus (i.e., feature) and the ASJC codes assigned to each paper (i.e., label). After that, based on similarity (which was calculated according to the ASJC classification model) and on procedures of previous studies [32,33], we assigned three ASJC codes to each national-funded research project; we described the probability of relevancy of each ASJC code that was assigned based on the title and abstract of the national-funded research project. Moreover, we applied the 95% threshold probability to improve our understanding over the relatedness between the assigned ASJC codes and the national-funded research projects. A conceptual diagram of this process is shown in Figure 2.

### 2.2. Co-Occurrence Matrix

To identify aging-related research fields from an interdisciplinary perspective, we adopted a co-occurrence technique. In text mining, the term “co-occurrence” refers to words that appear together in a sentence, paragraph, or text. Namely, the number of times that ASJC codes appear together in a project group reveals its relevance; the procedure is as follows:

- The co-occurrence matrix shows the number of times that the element *i* (from the first list) and the element *j* (from the second list) appear together in the text; namely, *i,j* = ASJC Codes.

Namely, the more often the ASJC codes appear in the projects, the higher the relevance of the projects that have these ASJC codes.

### 2.3. Clustering and Network Visualization

The network was built based on the number of appearances of ASJC codes in the projects, which was defined by the co-occurrence matrix. All nodes in the network were displayed based on the titles of the research fields present in the ASJC codes, and the font size was presented based on the frequency of the co-occurrence of each ASJC code in comparison with the other codes. By visualizing this network structure, we can figure out the relationship between the ASJC codes [33,34]. In this study, we used the modularity-based clustering method from VOSviewer (Leiden University, Leiden, The Netherlands, Version 1.16.15) [35]. We chose this methodology because a prior study showed that results yielded by the VOSviewer clustering technique are more precise and consistent than those yielded by k-means, which does not divide the largest cluster into smaller clusters (i.e., that have nodes which are closer to each other; see the comparison results of other clustering algorithms in supplementary Table S1 and Figure S1) [36]. The mapping and clustering were calculated based on minimizing (1) that explained in a prior study [37]. We describe the clustering algorithm herein:

(1) $$V\left(x_{1,}\ldots ,x_{n,}\right)=\;\underset{i<j}{\sum }\frac{2mc_{ij}}{c_{i}c_{j}}d_{ij}^{2}-\;\underset{i<j}{\sum }d_{ij}$$

where *n* = number of nodes in the network, *m* = number of links in the network, *c_ij_* = number of links between nodes *i* and *j*, *c_i_* = number of nodes *i*.

With respect to $x_{i}$, …, $x_{n}$; $d_{ij}$ = the distance between nodes *i* and *j*; in the case of mapping, it was calculated by the following formula:

(2) $$d_{ij}=\|x_{i}-x_{j}\|=\;\sqrt{\overset{p}{\underset{k=1}{\sum }}{\left(x_{ik}-x_{jk}\right)}^{2}}$$

where $x_{i}$ = vector denoting the location of node *i* in a *p*-dimensional map.

In the case of clustering, it was calculated by the following formula:

(3) dij={0 if xi= xj1γ if xi≠ xj

where $x_{i}$ = integer denoting the cluster to which node *i* belongs, *γ* = resolution parameter.

The resolution parameter (*γ* > 0) determines the level of detail of the clustering; the higher the value of the parameter, the larger the number of clusters produced. The range of the number of clusters went from 1 (*γ* = 0.1) to 6 (*γ* = 1.0). To conduct the semantic network analysis, we chose six clusters while considering the number and combination of items (i.e., ASJC codes) in individual clusters. Supposed that the number of cluster was five, health science that covers the 2700 fields (medicine area) and the 2900 fields (nursing area) of ASJC system and physical science that includes the 1700 fields (computer science) were gathered at the 5th cluster from the viewpoint of intermediate-level categories. When it was six, the cluster were divided into two clusters so that we could provide a strategic insight.

### 2.4. Defining Goals of Aging-Related Research Fields

**Figure 3:**
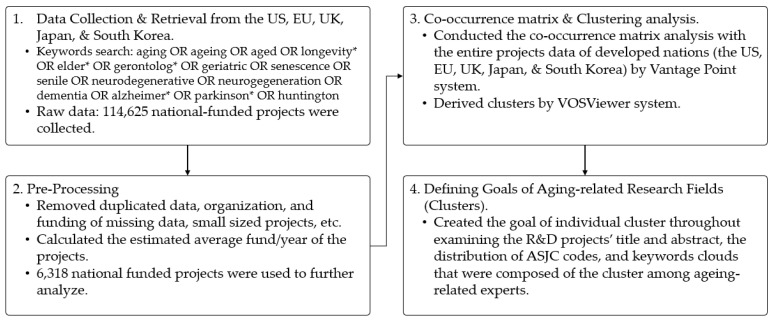
The workflow of data collection and analysis for aging-related national-funded research projects worldwide.

The definition of the goals for aging-related research fields can only be made by analyzing the title and abstract of the R&D projects, the distribution of the ASJC codes, and the keyword clouds—which comprise actual clusters of words that appeared throughout the discussions between experts on aging-related research, who can provide relevant knowledge and expertise for investigating particular research fields. Therefore, first, we assessed the approximate research fields by grasping the distribution of the ASJC codes and keyword clouds that constituted each cluster. Afterwards, we assessed the contents of the title and abstract of the R&D project in the clusters. Finally, we determined the goal of the research field of each cluster. The entire process is depicted in Figure 3.

## 3. Results

### 3.1. Interdisciplinary Aging-Related Research Fields of National-Funded Research Projects

**Figure 4:**
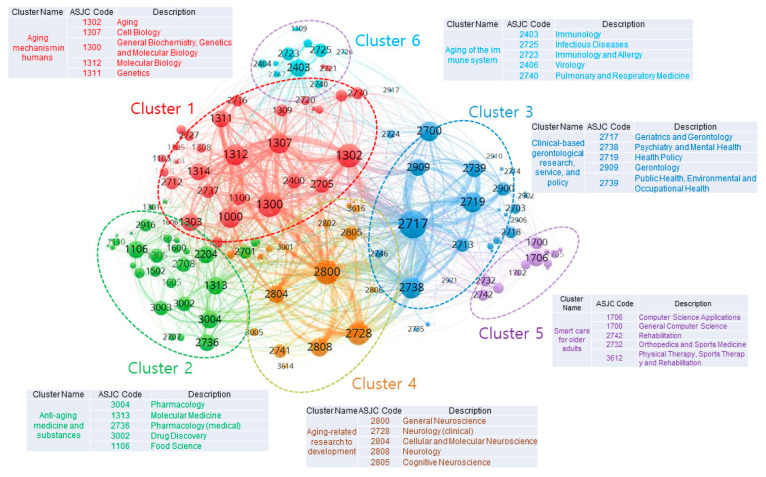
The interdisciplinary links between aging-related research fields.

The network visualization of aging-related research fields is shown in Figure 4. In this study, an item/node, which is treated as the object of interest, refers to the research fields (i.e., ASJC codes). A link, which implies a relationship between two items, refers to a co-occurrence link between research fields. The strength/weight of a link, in this study, indicates the number of projects in which two research fields appear together. The size of the label and the circle of a research field are determined by its weight; namely, the higher the weight of a research field, the larger its label and circles. The characteristics of a research field was determined by the cluster to which it belonged.

The aging-related research fields were divided into six clusters. After considering the titles and abstracts of the projects, their representative research fields, and the associated keywords, we created the ultimate goals that were pursued by each research field, and they are described as follows:

- Cluster 1. Cellular and molecular mechanisms of aging: Research on the identification of the biological mechanisms of aging; on the identification of the regulatory target genes related to aging and their mechanisms of action; on regenerative therapy using stem cells; and on the regulatory mechanisms of aging.
- Cluster 2. Anti-aging medicine and substances: Research on the diagnosis and treatment of infectious diseases by using advanced technology; on the development of anti-aging drugs (e.g., natural drugs, chemical synthetic drugs, biologic drugs, etc.); and on the development of high-value-added and functional food/drug materials derived from natural products.
- Cluster 3. Clinical-based research on aging-related diseases, medical services, and policies: Clinical research on the prevention, control, and treatment of aging-related diseases; on the rehabilitation of older adults; on aging-related public health; and on medical services for older adults.
- Cluster 4. Aging-related impairment of the brain and cognition: Basic, intermediary, clinical, and commercialization research on aging-related dementia, cognitive impairment, and neurodegenerative and mental health diseases.
- Cluster 5. Smart care for older adults: Research on the development of technology to improve the activities of daily living in older adults; and on the development of smart care platforms—using advanced technologies (e.g., robots, medical aids, artificial intelligence, and virtual reality (VR)/ augmented reality (AR))—for older adults.
- Cluster 6. Aging of the immune system: Research on the identification of the regulatory mechanisms of aging-related immune system decline; and on the enhancement of the immune defense system of older adults.

In the next subheadings, we describe the labeling processes and the detailed investigations for each cluster.

**Figure 5:**
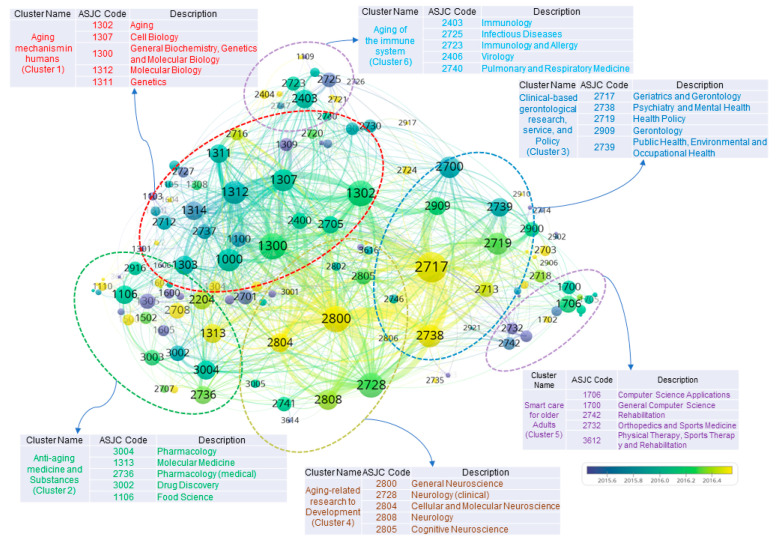
The interdisciplinary trend of changes in the aging-related research fields by period.

We present the trend of changes in the research fields by period in Figure 5. It shows that research fields, such as Geriatrics and Gerontology (2717), Psychiatry and Mental Health (2738), Epidemiology (2713), and Cellular and Molecular Neuroscience (2804), which are present in Clusters 3 and 4, have recently engaged in aging-related research.

#### 3.1.1. Cellular and Molecular Mechanisms of Aging (Cluster 1); Research on the Identification of the Biological Mechanisms of Aging; on the Identification of Target Genes Related to Aging and Their Mechanisms of Action; on Regenerative Therapy Using Stem Cells; and on the Regulatory Mechanisms of Aging

**Figure 6:**
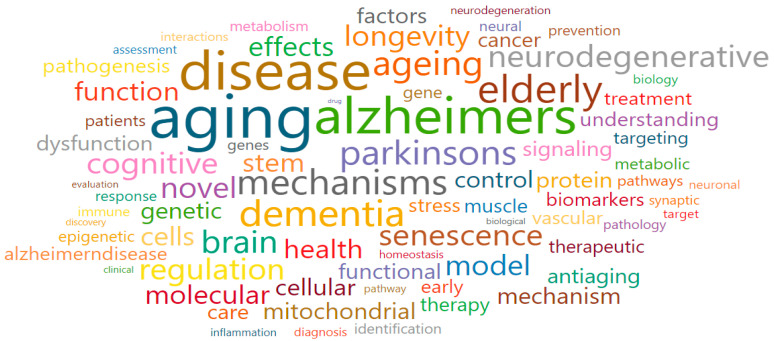
Words cloud in Cellular and molecular mechanisms of aging (Cluster 1).

Cellular and molecular mechanisms of aging (Cluster 1) comprised red nodes and was associated with a total of 31 research fields. The main research fields were Aging (1302), Cell biology (1307), General Biochemistry, Genetics and Molecular Biology (1300), Molecular Biology (1312), and Genetics (1311), as shown in Table 2. Moreover, some of the words that were associated with the keywords in Cluster 1 were the following: aging, dementia, elderly, senescence, neurodegenerative, mechanism, stem, cellular, molecular, longevity, brain, Alzheimer, Parkinson, and treatment (Figure 6). After reviewing the title and abstract of the projects, the main research fields, and the core keywords in Cluster 1, we confirmed that national-funded projects were mostly organized with the following goals (which also gave the name for the cluster): to explore molecular cell signaling mechanisms; discover regulatory target genes; develop genome-based biomarkers; and develop regenerative therapy using stem cells.

The main national-funded projects for Cluster 1 are shown in supplementary Table S2. For example, in the USA, the University of California San Diego was committed to spending approximately $6630 thousand per year between 2019–2021 on their study titled, Neurocognitive aging, MCI, and Alzheimer’s disease DNA methylation among diverse Latinos. In the EU, the Inserm (Institut national de la santé et de la recherche médicale) and the University of Leicester were committed to the following projects: Well-aging and the Tanycytic control of health (WATCH), with expenditures of $11,179 thousand per year between 2019–2025; and Telomere length measurement in UK Biobank: advancing understanding of biological ageing and age-related diseases, with costs of $2720 thousand per year between 2015–2020, respectively. In Asia (i.e., Japan and Korea), the Nagoya University and the Gwangju Institute of Science and Technology (GIST) were committed to the following projects: Brain protein aging and dementia control, with expenditures of $1436 thousand per year between 2014–2019; and Biological aging characterization study, with costs of $1289 thousand per year between 2015–2024, respectively.

#### 3.1.2. Anti-Aging Medicine and Substances (Cluster 2): Research on the Diagnosis and Treatment of Infectious Diseases by Using Advanced Technology; on the Development of Anti-Aging Drugs (e.g., Natural Drugs, Chemical Synthetic Drugs, Biologic Drugs, etc.); and on the Development of High-Value-Added and Functional Food/Drug Materials Derived from Natural Products

**Figure 7:**
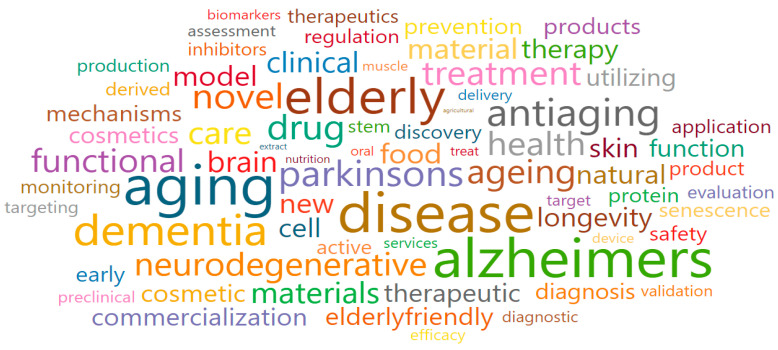
Words cloud in Anti-aging medicine and substances (Cluster 2).

Anti-aging medicine and substances (Cluster 2) comprised green nodes and was associated with a total of 31 research fields. The main research fields were Pharmacology (3004), Molecular Medicine (1313), Pharmacology (medical) (2736), Drug Discovery (3002), and Food Science (1106), as shown in Table 3. Moreover, some of the words that were associated with the keywords of Cluster 2 were the following: aging, dementia, elderly, senescence, antiaging, drug, food, discovery, natural, materials, treatment, therapeutic, diagnosis, model, clinical, cosmetic, and commercialization (Figure 7). After a review procedure equal to that in Cluster 1, we confirmed that national-funded projects were mostly organized with the following goals (which also gave the name for the cluster): to discover anti-aging ingredients that can help improve aging-related diseases; develop anti-aging treatments; develop natural or herbal medicine-based materials; develop high-value-added and functional food/drug materials derived from natural products for older adults.

The main national-funded projects for Cluster 2 are shown in supplementary Table S3. For example, in the USA, the Emory University was committed to spending around $7499 thousand per year between 2019–2024 on their study titled, Open drug discovery center for Alzheimer’s disease (Open-AD). In the EU, the Netherlands Cancer Institute (Stichting Het Nederlands Kanker Instituut-Antoni Van Leeuwenhoekziekenhuis) and the University College London were committed to the following projects: Senescence therapy for cancer, with expenditures of $2809 thousand per year between 2018–2023; and Gene therapy for Childhood Parkinsonism: Dopamine transporter deficiency syndrome, with costs of $652 thousand per year between 2018–2020, respectively. In Asia, the University of Tokyo and the Korea Research Institute of Bioscience and Biotechnology (KRIBB) were committed to the following projects: Development of a novel antiaging strategy by elucidating the mechanisms regulating aging through a muscle centric organ network, with expenditures of $1819 thousand per year between 2015–2020; and Agricultural biological micro-biome based material for well-aging innovation development and commercialization, with costs of $1377 thousand per year between 2019–2023, respectively.

#### 3.1.3. Clinical-Based Research on Aging-Related Diseases, Medical Services, and Policies (Cluster 3): Clinical Research on the Prevention, Control, and Treatment of Aging-Related Diseases; on the Rehabilitation of Older Adults; on Aging-Related Public Health; and on Medical Services for Older Adults

**Figure 8:**
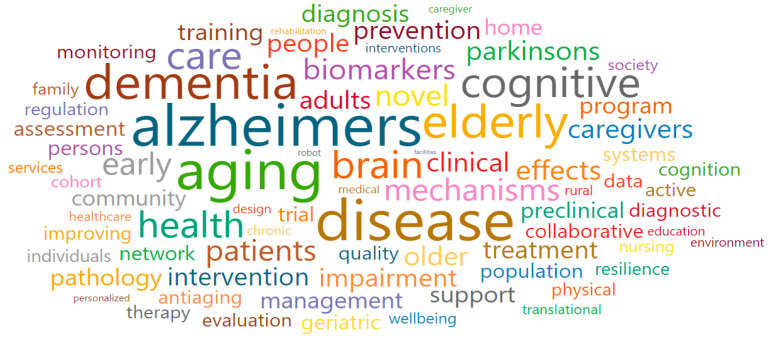
Words cloud in Clinical-based research on aging-related diseases, medical services, and policies (Cluster 3).

Clinical-based research on aging-related diseases, medical services, and policies (Cluster 3) comprised blue nodes and was associated with a total of 27 research fields. The main research fields were Geriatrics and Gerontology (2717), Psychiatry and Mental Health (2738), Health Policy (2719), Gerontology (2909), and Public Health, Environmental and Occupational Health (2739), as shown in Table 4. Moreover, some of the words that were associated with the keywords of Cluster 3 were the following: aging, dementia, elderly, senescence, care, cognitive, prevention, caregivers, health, home, patients, clinical, preclinical, monitoring, support, management, community, and collaborative (Figure 8). After a review procedure equal to that for Cluster 1, we confirmed that national-funded projects were mostly clinical research that were organized with the following goals (which also gave the name for the cluster): to develop health promotion initiatives for older adults; and to manage chronic diseases through health status improvement and rehabilitation.

The main national-funded projects for Cluster 3 are shown in supplementary Table S4. For example, in the USA, the University of California, Davis, was committed to spending around $12,173 thousand per year between 2017–2022 on their study titled, Epidemiology of age-related dementia, mild cognitive impairment, and brain pathology in a multiethnic cohort of oldest-old. In the EU, the University of Zurich and the University of Sussex were committed to the following projects: VitaminD3-Omega3-Home Exercise-HeALTHy aging and longevity trial (DO-HEALTH), with expenditures of $14,807 thousand per year between 2012–2017; and Determinants of quality of life, care and costs, and consequences of inequalities in people with dementia and their carers (DETERMIND), with costs of $4986 thousand per year between 2019–2023, respectively. In Asia, the Kyoto University and the Korea Institute of Science and Technology (KIST) were committed to the following projects: Lifestyle and brain function: Inquiry in psychological science into successful aging, with expenditures of $1204 thousand per year between 2016–2021; and The elderly disabled target daily disability prevention and overcoming technological development, with costs of $2117 thousand per year between 2019–2027, respectively.

#### 3.1.4. Aging-Related Impairment of the Brain and Cognition (Cluster 4): Basic, Intermediary, Clinical, and Commercialization Research on Aging-Related Dementia, Cognitive Impairment, and Neurodegenerative and Mental Health Diseases

**Figure 9:**
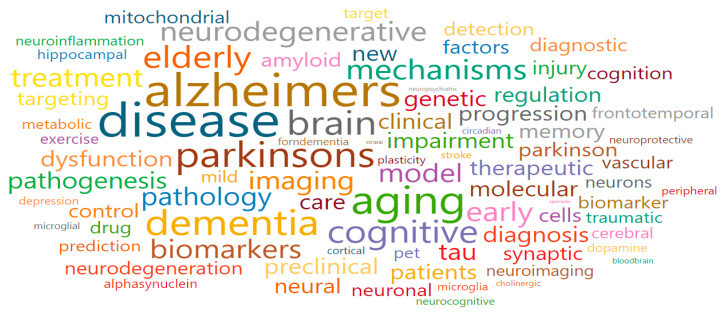
Words cloud in Aging-related impairment of the brain and cognition (Cluster 4).

Aging-related impairment of the brain and cognition (Cluster 4) comprised yellow nodes and was associated with a total of 17 research fields. The main research fields were General Neuroscience (2800), Neurology (clinical) (2787), Cellular and Molecular Neuroscience (2804), Neurology (2808), and Cognitive Neuroscience (2805), as shown in Table 5. Moreover, some of the words that were associated with the keywords of Cluster 4 were the following: aging, dementia, Alzheimer, Parkinson, elderly, brain, cognitive, tau, neuronal, impairment, neurodegenerative, mechanisms, clinical, pathology, diagnosis, biomarker, and treatment (Figure 9). After a review procedure equal to that in Cluster 1, we confirmed that national-funded projects were mostly organized to develop full-cycle R&D activities related to brain and cognitive diseases—a goal that also gave the name for the cluster; for example, basic, intermediary, clinical, and commercialization research on neurodegenerative and aging-related mental health diseases.

The main national-funded projects for Cluster 4 are shown in supplementary Table S5. For example, in the USA, the Mayo Clinic was committed to spending $10,639 thousand per year between 2017–2022 on their study titled, Prevention of Alzheimer’s disease in women: risks and benefits of hormone therapy. In the EU, the Stichting VUmc and the University College London were committed to the following projects: Amyloid imaging to prevent Alzheimer’s disease (AMYPAD), with expenditures of $30,967 thousand per year between 2016–2021; and The UK genetic frontotemporal dementia initiative (GENFI), with costs of $3441 thousand per year between 2015–2020, respectively. In Asia, the Niigata University and the Korea Institute of Science and Technology (KIST) were committed to the following projects: Approach to neurodegenerative disorders by elucidation of the excretion pathway of the brain, with expenditures of $414 thousand per year between 2019–2022; and Older generations predict Alzheimer’s early treatments and patient care technology, with costs of $4975 thousand per year between 2015–2021, respectively.

#### 3.1.5. Smart Care for Older Adults: Research on the Development of Technology to Improve the Activities of Daily Living in Older Adults; and on the Development of Smart Care Platforms—Using Advanced Technologies (e.g., Robots, Medical Aids, Artificial Intelligence, and VR/AR)—For Older Adults

**Figure 10:**
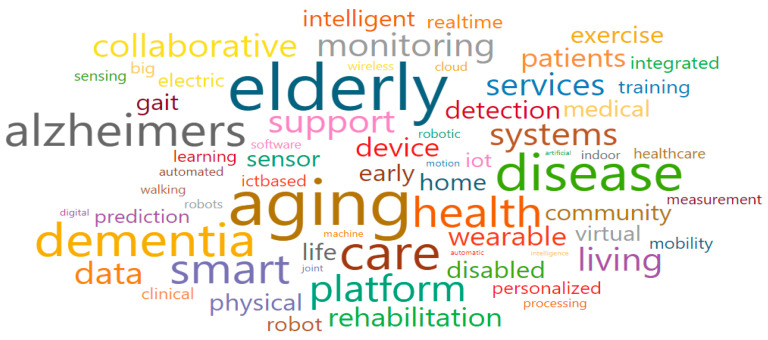
Words cloud in Smart care for older adults (Cluster 5).

Smart care for older adults (Cluster 5) comprised purple nodes and was associated with a total of 12 research fields. The main research fields were Computer Science Applications (1706), General Computer Science (1700), Rehabilitation (2742), Orthopedics and Sports Medicine (2732), and Physical Therapy, Sports Therapy and Rehabilitation (3612), as shown in Table 6. Moreover, some of the words that were associated with the keywords of Cluster 5 were the following: aging, dementia, elderly, intelligent, wearable, virtual, platform, smart, data, device, detection, robot, prediction, sensor, IoT (i.e., Internet of Things), home, and rehabilitation (Figure 10). After a review procedure equal to that in Cluster 1, we confirmed that national-funded projects were mostly organized with the following goals (which also gave the name for the cluster): to develop robots that support the daily life of older adults; improve the older adult-friendly smart residential environment; and create a virtual space which can complement the physical functions of older adults.

The main national-funded projects for Cluster 5 are shown in supplementary Table S6. For example, in the USA, the University of Michigan was committed to spending around $3475 thousand per year between 2018–2023 on their study titled, Rehabilitation research and training center (RRTC) on promoting healthy aging for people with long-term physical disabilities. In the EU, the Charité-Universitätsmedizin Berlin and the Heriot-Watt University were committed to the following projects: Personalized recommendations for neurodegenerative disease, with expenditures of $17,015 thousand per year between 2018–2022; and Ageing well in urban environments: developing age friendly cities and communities, with costs of $471 thousand per year between 2018–2020, respectively. In Asia, the Tokushima University and the Electronics and Telecommunications Research Institute (ETRI) were committed to the following projects: Development of the method of nursing as caring for the elderly by collaborating with humanoid interactive robot, with expenditures of $382 thousand per year between 2017–2022; and Room environment human care robotic technology to cope with aging society, with costs of $5022 thousand per year between 2017–2021, respectively.

#### 3.1.6. Aging of the Immune System: Research on the Identification of the Regulatory Mechanisms of Aging-Related Immune System Decline; and on the Enhancement of the Immune Defense System of Older Adults

**Figure 11:**
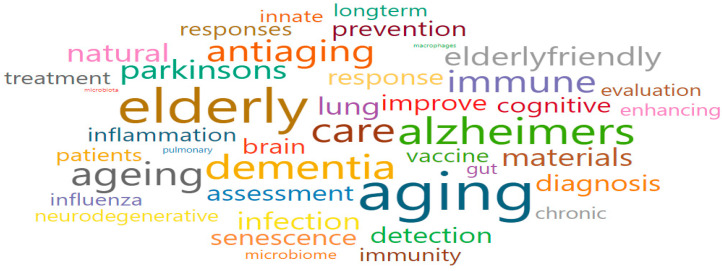
Words cloud in Aging of the immune system (Cluster 6).

Aging of the immune system (Cluster 6) comprised turquoise nodes and was associated with a total of 11 research fields. The main research fields were Immunology (2403), Infectious Diseases (2725), Immunology and Allergy (2723), Virology (2406), and Pulmonary and Respiratory Medicine (2740), as shown in Table 7. Moreover, some of the words that were associated with the keywords of Cluster 6 were the following: aging, dementia, elderly, immune, immunity, influenza, innate, infection, lung, prevention, enhancing, and inflammation (Figure 11). After a review procedure equal to that in Cluster 1, we confirmed that national-funded projects were mostly organized with the following goals (which also gave the name for the cluster): to identify the aging-related mechanism that causes dysfunctions in the immune response system; and to strengthen the immune defense system of older adults.

The main national-funded projects for Cluster 6 are shown in supplementary Table S7. For example, in the USA, the Regents of the University of California was committed to spending around $7375 thousand per year between 2015–2020 on their study titled, Establishment to senescence: Plant-microbe and microbe-microbe interactions mediate switchgrass sustainability. In the EU, the Universitair Medisch Centrum Utrech and the King’s College London were committed to the following projects: Vaccines and infectious diseases in the ageing population (VITAL), with expenditures of $14,081 thousand per year between 2019–2023; and Multi-scale analysis of B cell responses in ageing (MABRA), with costs of $2351 thousand per year between 2014–2016, respectively. In Asia, the Osaka University and the Korea University were committed to the following projects: Epidemiological analyses for the impact of bacterial and virus infection to the occurrence of dementia by long-term cohort studies, with expenditures of $400 thousand per year between 2018–2022; and Geriatric treatment target excavation of severe respiratory inflammation and control research, with costs of $270 thousand per year between 2017–2022, respectively.

### 3.2. Regional/National Differences on Aging-Related Funded Projects

The representative aging-related national-funded projects of the analyzed regions are shown in Table 8. The USA projects ranged from basic research (e.g., investigating the underlying mechanisms of aging-related brain diseases) to research on the identification of preclinical therapies and on therapeutic targets. In the EU and UK, projects were mostly related to their general regional goal of promoting active aging; thus, they ranged from studies on the development of high-value-added and functional food/drug materials derived from natural products, to studies on infectious diseases and immunity for older adults, and to studies on diagnosis and treatment of aging-related dementia. In Japan, the projects focused on the promotion of anti-aging through muscle strength and physical performance improvement in older adults, and on the daily life of older adults with aging-related mental disorders. In Korea, projects focused on the early detection, diagnosis, and prediction of Alzheimer’s disease, and on the management of aging-related chronic diseases; these were mostly translational research, namely, aiming to bridge the gap between basic and clinical research.

## 4. Discussion

The current (super-)aging society issues that we have been experiencing are not a problem that relate only to a single nation; instead, they are a global problem. As demonstrated, many nations have launched multiple initiatives and endeavored to build an international R&D collaboration strategy to deal with a global aging society. Overall, this study aimed to create a practical and coherent approach to assist in the decision-making processes related to international R&D collaboration strategies/programs for aging-related issues.

First, we endeavored to classify the research fields and the goals of the R&D projects. As remarked, various nations demonstrated their willingness to solve aging-related issues by conducting various national-funded research projects; we classified the research fields of these projects based on the ASJC codes. Then, by combining the understanding of the content of the projects (i.e., of their title and abstract), the representative research fields for the projects, and the keywords that were associated with the clusters, we were able to grasp the ultimate goals of specific R&D projects. We categorized their goals into six clusters.

The second step was to provide information about the status of aging-related national-funded projects across regions by clusters. This information may allow policy-makers and R&D program designers to understand which projects can be conducted domestically or abroad, thereby allowing for them to make more well-informed decisions regarding the current scientific capabilities of individual research fields. In the USA, the NIA was shown to play a central role in the associations between public and private research institutes; these included the Buck Institute, the Banner Alzheimer’s Institute, Harvard University, and Stanford University. Specifically, the NIA has invested not only on basic scientific research regarding the mechanisms and characteristics of aging but also on anti-aging projects for controlling and treating aging-related diseases. In Japan, the National Center for Geriatrics and Gerontology (NCGG) was the one playing a central role in encouraging disciplinary convergence—and the development of interdisciplinary projects—to thereby foster the anti-aging industry and scientific fields; they did so by collaborating with clinical-oriented organizations (i.e., geriatric hospitals, special hospitals for the treatment of dementia, special hospitals for the treatment of periodontal diseases, institutes for aging research, and centers for social research on aging).

In the EU and the UK, various policies (e.g., the EIP on AHA, Silver Economy, etc.) will be initiated under the Horizon 2020, the biggest EU Research and Innovation programme with nearly €80 billion of funding available over 7 years (2014 to 2020), aiming to promote active and healthy aging for people living in Europe; accordingly, the EU and the UK have strategically invested in the health and biomedical fields by conducting multinational consortium projects. For example, research on the development and application of high-value-added and functional food/drug materials derived from natural products. Finally, in Korea, most projects were basic research on aging-related diseases (e.g., cancer, dementia, and cardiovascular diseases) and on aging-related changes in organs (e.g., musculoskeletal organs, skin, and stem cells); nonetheless, to improve the national drug development capacity, the government has recently shifted its investment strategy toward endowing translational research.

The final step was to highlight the potential use of the aforementioned information as a framework for stakeholders—from a wide variety of governmental and academic institutions and industries—to coordinate the development of novel aging-related project programs both domestically and/or internationally, or even to manage the existing ones. Our results yielded multiple sources of information regarding aging-related R&D projects, which include the following: the research organizations that conducted the studies; the specific goals of the projects (i.e., project title); their project periods; and their funding. We hope that these serve as appropriate data to conduct stakeholders in the right direction during future R&D strategy/program development; this may be achieved by using our data to bridge the gap between stakeholders who have conflicting interests, different perspectives, and different ways of understanding the same topic during their strategical discussions. For example, regarding aging-related dementia, the Korean government may be able to establish a strategy that allows for international advanced research organizations (e.g., those in the USA and the EU) to not only access complementary resources present only in Korea but also examine the aging-related diseases that have high levels of prevalence in Korean older adults. Moreover, our results demonstrated the need to build a wide range of databases related to biometric information (i.e., on genomes, transcriptomes, proteomes, metabolomes, epigenomes, and lipidomes that appeared as being related to natural process of aging) at a global scale; such databases may enable for an improved diagnosis of aging-related diseases, discovery of therapeutic targets, and management of proactive aging at the international level. Namely, to leverage the individual national capabilities, we see the need for further international discussions regarding practical, scientifical, and technological policies and regarding R&D strategies and collaboration.

Although the world currently acknowledges the importance of aging-related scientific discussions and technological development, the actual prioritization of these topics remain undermined by other, more urgent topics (e.g., infectious diseases that have been occurring worldwide). Recently, the World Health Organization (WHO) classified aging itself as a disease [38], thus recognizing aging as a health factor that can be directly prevented and treated and concomitantly creating a research-friendly environment for anti-aging drugs. Based on our findings and the studies analyzed, the development of common, international, and standardized markers may prove to be a prerequisite for the effective management and treatment of aging. This could be operationalized by stakeholders endeavoring to develop a global aging-related research database; one that allows for researchers to track changes in a variety of biometric information in the context of natural aging. Although this can only be achieved by the establishment of closer international collaborations, such a facilitating environment could stimulate the development of anti-aging markers for humans. Furthermore, we see the necessity of setting up global policies to foster exchange programs among advanced researchers based on collaboration, and these should stretch across all types of research (i.e., from basic to translational research).

## 5. Conclusions

Based on prior research that describes the role of data saliency, credibility, and legitimacy [27], we believe that this study may facilitate the association between knowledge and action in the R&D strategy development process by providing usable data that have these three characteristics. In this study, we ensured data saliency by the degree of relevant information that we can offer to decision-making stakeholders; specifically, we describe up-to-date and correlated data on various characteristics of global national-funded research projects (including on funding), and these data are normally very hard to be deduced when a scientific publication or patent-based analysis in undertaken. We ensured data credibility by providing enough data on the projects (i.e., we combined data collected from databases operated by the analyzed governments with the systematic procedures of classification and labeling) to allow for the conduction of peer reviews. Finally, we ensured data legitimacy by using a methodology that minimizes the potential for bias and ensures greater equity regarding participation (i.e., we addressed the following major questions of strategy planning during the discussion: Where are we going? Where are we now? And how can we get there?).

Despite the numerous contributions, this study also has limitations that present some challenging questions for future research. One inherent limitation concerns the USA dataset, which ranked R&D projects by their expenditure, not by country. Additionally, the Korean NTIS is a centralized database that encompasses the entire national data on funding, so it differs from the databases in the USA, EU, UK, and Japan. Therefore, it leads to a “home advantage” effect, which may have led to the underestimation of the R&D activities of organizations operating outside of Korea—mainly owing to the massive scale of the domestic data on funding. We suggest for future studies to collect more funding data from individual member states of the EU and Japan, as well as to conduct a comparative analysis per cluster among nations regarding the absolute amount of R&D funding; future studies with such a methodology can support the future establishment of an aging-related R&D project portfolio management among these nations. We focused on aging-related R&D projects in this study because they were recognized as a solution to reduce the various challenges around the world. However, the proposed approach may be applied to the narrow aging-related social issues with collecting documents that have political, law, psychological, and urban-related contents for older adults.

## Figures and Tables

**Table 1:** Aging and aging-related national-funded project data and search terms.

Search Terms	Regions	Amount of Raw Data	Number of Data Utilized
aging OR ageing OR aged OR longevity * OR elder * OR gerontology * OR geriatric OR senescence OR senile OR neurodegenerative OR neurodegeneration OR dementia OR Alzheimer * OR Parkinson * OR Huntington *	United States of America (USA)	87,715	4273
	European Union (EU)	2385	403
	United Kingdom (UK)	4066	389
	Japan (JP)	6247	275
	South Korea (KR)	14,211	978
	Total (2012—2019)	114,625	6318

The asterisk (*) in search term is used to broaden a search by finding words that start with the same letters.

**Table 2:** Major research fields in Cellular and molecular mechanisms of aging (Cluster 1).

Cluster Name	Research Field (ASJC)	Description	Frequency
Cellular and molecular mechanisms of aging (Cluster 1)	1302	Aging	867
	1307	Cell Biology	726
	1300	General Biochemistry, Genetics and Molecular Biology	683
	1312	Molecular Biology	627
	1311	Genetics	357

**Table 3:** Major research fields in Anti-aging medicine and substances (Cluster 2).

Cluster Name	Research Fields (ASJC)	Description	Frequency
Anti-aging medicine and substances (Cluster 2).	3004	Pharmacology	246
	1313	Molecular Medicine	210
	2736	Pharmacology (medical)	147
	3002	Drug Discovery	135
	1106	Food Science	128

**Table 4:** Major research fields in Clinical-based research on aging-related diseases, medical services, and policies (Cluster 3).

Cluster Name	Research Fields (ASJC)	Description	Frequency
Clinical-based research on aging-related diseases, medical services, and policies (Cluster 3)	2717	Geriatrics and Gerontology	1979
	2738	Psychiatry and Mental Health	934
	2719	Health Policy	539
	2909	Gerontology	396
	2739	Public Health, Environmental and Occupational Health	299

**Table 5:** Major research fields in Aging-related impairment of the brain and cognition (Cluster 4).

Cluster Name	Research Fields (ASJC)	Description	Frequency
Aging-related impairment of the brain and cognition (Cluster 4)	2800	General Neuroscience	2059
	2728	Neurology (clinical)	850
	2804	Cellular and Molecular Neuroscience	683
	2808	Neurology	439
	2805	Cognitive Neuroscience	254

**Table 6:** Major research fields in Smart care for older adults (Cluster 5).

Cluster Name	Research Fields (ASJC)	Description	Frequency
Smart care for older adults (Cluster 5)	1706	Computer Science Applications	122
	1700	General Computer Science	97
	2742	Rehabilitation	71
	2732	Orthopedics and Sports Medicine	56
	3612	Physical Therapy, Sports Therapy and Rehabilitation	51

**Table 7:** Major research fields in Aging of the immune system (Cluster 6).

Cluster Name	Research Fields (ASJC)	Description	Frequency
Aging of the immune system (Cluster 6)	2403	Immunology	185
	2725	Infectious Diseases	80
	2723	Immunology and Allergy	70
	2406	Virology	43
	2740	Pulmonary and Respiratory Medicine	26

**Table 8:** The representative national-funded research projects by region, funding, start and end dates, and research field.

Region	Organization	Project Title	Estimated Average Fund/Year (US $1000)	Start Date	End Date	Research Field
USA	Banner Alzheimer’s Institute	Alzheimer’s prevention initiative	15,253	2012-05-18	2017-04-30	2719;2738;2713
USA	University of Southern California	Combination antiamyloid therapy for preclinical Alzheimer’s disease	4186	2018-07-15	2023-06-30	2403
USA	Stanford University	Regulation of immune cell metabolism in aging and Alzheimer’s disease role of the kynurenine pathway	2043	2017-09-15	2022-06-30	1307;1302
EU	Eberhard Karls University of Tübingen	Integrated European omics research project for diagnosis and therapy in rare neuromuscular and neurodegenerative diseases	19,088	2012-10-01	2017-09-30	1303;1311;1312
EU	Universitaet Zuerich	VitaminD3-Omega3-Home Exercise-heaLTHy aging and longevity trial (DO-HEALTH),	14,807	2012-01-01	2017-06-30	2700;2719;2739
EU	Universitair Medisch Centrum Utrecht	Vaccines and infectious diseases in the ageing population	14,081	2019-01-01	2023-12-31	2403;2725
UK	University of Oxford	UKDP: Integrated DEmentiA research environment idea (IDEA)	8975	2015-04-01	2015-08-14	2800
UK	Newcastle University	Centre for ageing and vitality	3339	2014-07-01	2019-06-30	2916
UK	King’s College London	Multi-scale analysis of B cell responses in ageing (MABRA)	2351	2014-05-01	2016-04-30	2403;2723
Japan	The University of Tokyo	Development of a novel antiaging strategy by elucidating the mechanisms regulating aging through a muscle centric organ network	1819	2015-05-29	2020-03-31	1305
Japan	Kyoto University	Lifestyle and brain function inquiry in psychological science into successful aging	1204	2016-05-31	2021-03-31	2909;2717
Japan	Tokyo Metropolitan Geriatric Hospital and Institute of Gerontology	Effect of vitamin C deficiency on the fetal growth and aging	163	2012-04-01	2015-03-31	2701
Korea	Korea Institute of Science and Technology	Older generations predict Alzheimer’s early treatments and patient care technology	4975	2015-12-01	2021-11-30	2728
Korea	Korea Research Institute of Bioscience and Biotechnology	Age-related chronic diseases aging corresponding source control technology	1463	2012-01-01	2014-12-31	1303;1312
Korea	Gyeongsang National University	Antiaging biomedical science research center	1038	2015-10-01	2022-02-28	1302;1307;1312

## Supplementary Materials

The following are available online at https://www.mdpi.com/1660-4601/17/22/8545/s1, Table S1: The results of comparison between modularity and k-means clustering, Table S2: Cellular and molecular mechanisms of aging (Cluster 1), Table S3: Anti-aging medicine and substances (Cluster 2), Table S4: Clinical-based research on aging-related diseases, medical services, and policies (Cluster 3), Table S5: Aging-related impairment of the brain and cognition (Cluster 4), Table S6: Smart care for older adults (Cluster 5). Table S7: Aging of the immune system (Cluster 6), Figure S1: The curve of the total within-cluster sum of square according to the number of clusters *k*.
