# Supplementary material for: International R&D Collaboration for a Global Aging Society: Focusing on Aging-Related National-Funded Projects
Source: Int J Environ Res Public Health. 2020 Nov 18;17(22):8545. doi: 10.3390/ijerph17228545 (PMC7698711; doi:10.3390/ijerph17228545)
Supplement: Supplementary file 1 [file ijerph-17-08545-s001.pdf]

The optimal number of clusters and the comparison between modularity and k-means clustering

In this study, we conducted the k-means algorithm to specify the number of clusters and to compare the modularity clustering technique. In order to determine optimal clusters, the elbow method was used. The plotting image of the curve of the total within-cluster sum of square according to the number of clusters *k* was shown as follows:

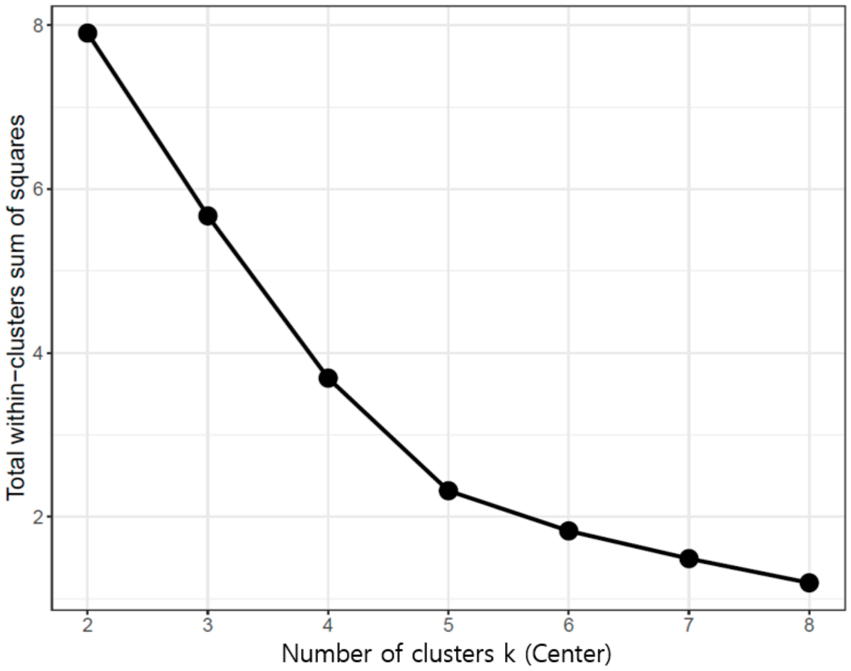

Figure S1. The curve of the total within-cluster sum of square according to the number of clusters *k*.

The result suggests that five or six is the optimal number of clusters as it appears to be the bend in the elbow. The results of comparison between modularity and k-means clustering as follows:

Table S1. The results of comparison between modularity and k-means clustering

NO.	Label (ASJC)	Modularity clustering		k-means clustering						
		cluster_5	cluster_6	center_2	center_3	center_4	center_5	center_6	center_7	center_8
1	1000	2	1	1	1	1	3	6	7	7
2	1100	2	1	1	3	3	3	6	6	8
4	1102	3	2	1	3	3	5	3	3	6
5	1103	2	1	1	3	3	5	5	5	5
7	1105	2	1	1	3	3	5	3	3	6
8	1106	3	2	1	1	1	3	6	7	7
9	1107	3	2	1	3	4	4	4	4	4
10	1108	3	2	1	3	4	4	4	4	4
11	1109	5	6	1	3	3	5	5	5	5
12	1110	3	2	1	3	4	4	4	3	3
13	1111	3	2	1	3	3	5	3	3	3
14	1300	2	1	2	1	1	1	1	1	1

NO.	Label (ASJC)	Modularity clustering		k-means clustering						
		cluster_5	cluster_6	center_2	center_3	center_4	center_5	center_6	center_7	center_8
15	1301	3	2	1	3	3	5	5	5	6
16	1302	2	1	2	1	1	1	1	1	1
17	1303	2	1	1	1	1	3	6	7	7
18	1304	2	1	1	3	4	4	3	3	3
19	1305	3	2	1	3	3	3	6	6	8
20	1306	2	1	1	3	3	3	3	6	8
21	1307	2	1	2	1	1	1	1	1	1
22	1308	2	1	1	3	3	4	3	3	3
23	1309	2	1	1	3	3	5	5	6	6
24	1310	2	1	1	3	3	5	3	3	6
25	1311	2	1	1	1	1	3	6	7	7
26	1312	2	1	2	1	1	1	1	1	1
27	1313	3	2	1	1	1	3	6	7	7
28	1314	2	1	1	1	1	3	6	7	7
30	1500	3	2	1	3	4	4	3	3	3
31	1502	3	2	1	3	3	3	3	6	8
32	1503	3	2	1	3	3	5	3	3	3
36	1600	3	2	1	3	3	5	5	5	6
37	1602	3	2	1	3	4	4	4	4	3
38	1603	3	2	1	3	4	4	3	3	3
39	1604	2	1	1	3	4	4	4	3	3
40	1605	3	2	1	3	3	5	3	6	6
41	1606	2	1	1	3	3	5	5	5	5
42	1607	3	2	1	3	3	5	5	5	5
43	1700	1	5	1	3	3	3	6	6	8
44	1702	1	5	1	3	4	4	4	4	4
45	1704	1	5	1	3	3	5	3	3	3
46	1705	1	5	1	3	3	5	3	3	3
47	1706	1	5	1	1	1	3	6	7	8
49	1708	1	5	1	3	3	5	3	3	3
50	1709	1	5	1	3	3	5	3	6	6
51	1710	1	5	1	3	4	4	3	3	3
53	1712	1	5	1	3	3	5	3	3	3
73	2204	3	2	1	1	1	3	6	7	7
95	2400	2	1	1	3	3	3	6	6	8
96	2402	3	2	1	3	3	5	5	5	6
97	2403	5	6	1	1	1	3	6	7	7
98	2404	5	6	1	3	4	4	4	4	4
99	2405	5	6	1	3	4	4	4	4	3
100	2406	5	6	1	3	3	5	3	3	6
114	2700	1	3	1	1	1	3	6	7	7
115	2701	3	2	1	3	3	3	6	6	8
116	2702	4	4	1	3	3	5	3	3	3
117	2703	1	3	1	3	4	4	3	3	3

NO.	Label (ASJC)	Modularity clustering		k-means clustering						
		cluster_5	cluster_6	center_2	center_3	center_4	center_5	center_6	center_7	center_8
118	2705	2	1	1	1	1	3	6	7	8
119	2706	1	3	1	3	4	4	4	4	4
120	2707	3	2	1	3	4	4	3	3	3
121	2708	3	2	1	3	4	3	6	6	8
122	2711	1	3	1	3	3	5	3	3	6
123	2712	2	1	1	3	3	3	6	6	8
124	2713	1	3	1	3	1	3	6	6	8
125	2714	1	3	1	3	3	5	5	5	6
126	2715	2	1	1	3	4	4	4	3	3
127	2716	2	1	1	3	4	3	6	6	8
128	2717	1	3	2	2	2	2	2	2	2
129	2718	1	3	1	3	4	3	3	6	8
130	2719	1	3	2	1	1	1	1	1	1
131	2720	2	1	1	3	4	4	3	3	3
132	2721	2	1	1	3	4	4	4	3	3
133	2722	3	2	1	3	4	4	4	4	4
134	2723	5	6	1	3	3	3	6	6	8
135	2724	1	3	1	3	4	4	4	3	3
136	2725	5	6	1	3	3	3	6	6	8
137	2726	5	6	1	3	3	5	5	5	5
138	2727	2	1	1	3	3	5	3	6	6
139	2728	4	4	2	1	1	1	1	1	1
140	2729	2	1	1	3	3	5	3	5	6
141	2730	2	1	1	3	3	3	3	6	8
142	2731	4	4	1	3	3	5	5	5	6
143	2732	1	5	1	3	3	5	5	5	6
144	2733	4	4	1	3	3	5	5	5	5
145	2734	3	2	1	3	4	4	4	3	3
146	2735	1	3	1	3	4	4	4	4	4
147	2736	3	2	1	1	1	3	6	7	7
148	2737	2	1	1	3	3	3	6	6	8
149	2738	1	3	2	1	1	1	1	1	1
150	2739	1	3	1	1	1	3	6	7	7
151	2740	5	6	1	3	3	5	3	3	3
152	2741	4	4	1	3	3	3	6	6	8
153	2742	1	5	1	3	3	5	3	6	6
154	2743	2	1	1	3	3	5	5	5	5
155	2745	5	6	1	3	4	4	4	3	3
156	2746	1	3	1	3	3	5	3	3	6
157	2747	5	6	1	3	3	5	3	3	6
158	2748	2	1	1	3	3	5	3	3	6
159	2800	4	4	2	2	2	2	2	2	2
161	2802	4	4	1	3	3	5	3	3	6
162	2803	1	3	1	3	3	5	5	5	5

NO.	Label (ASJC)	Modularity clustering		k-means clustering						
		cluster_5	cluster_6	center_2	center_3	center_4	center_5	center_6	center_7	center_8
163	2804	4	4	2	1	1	1	1	1	1
164	2805	4	4	1	1	1	3	6	7	8
165	2806	4	4	1	3	4	4	4	3	3
167	2808	4	4	2	1	1	1	1	7	7
168	2809	4	4	1	3	3	5	5	5	6
169	2900	1	3	1	3	3	3	6	6	8
170	2902	1	3	1	3	3	5	3	5	6
171	2905	1	3	1	3	4	4	4	4	3
172	2906	1	3	1	3	4	4	3	3	3
173	2907	1	3	1	3	3	5	5	5	5
174	2909	1	3	1	1	1	3	6	7	7
175	2910	1	3	1	3	4	4	4	4	4
176	2911	1	3	1	3	3	5	5	5	5
177	2912	4	4	1	3	4	4	4	4	4
178	2914	1	3	1	3	3	5	5	5	5
179	2916	3	2	1	3	3	3	6	6	8
180	2917	2	3	1	3	4	4	4	3	3
182	2921	1	3	1	3	3	5	3	3	6
183	3000	3	2	1	3	4	4	4	3	3
184	3001	4	4	1	3	4	4	4	4	4
185	3002	3	2	1	3	3	3	6	6	8
186	3003	3	2	1	3	3	3	6	6	8
187	3004	3	2	1	1	1	3	6	7	7
188	3005	4	4	1	3	3	5	3	3	3
204	3601	3	2	1	3	3	5	5	5	5
206	3611	1	3	1	3	4	4	4	3	3
207	3612	1	5	1	3	3	5	3	6	6
208	3614	4	4	1	3	3	5	5	5	6
209	3616	4	4	1	3	3	5	3	3	6

Table S2. Cellular and molecular mechanisms of aging (Cluster 1)

Region	Organization	Project Title	Estimated Average Fund/Year (US$1,000)	Start Date	End Date	Research field (ASJC)
USA	University of Pennsylvania	Consortium for Alzheimer's sequence analysis casa	12,639	2014-06-15	2019-05-31	1311;2716;1312
USA	University of California San Diego	Neurocognitive aging, MCI, and Alzheimer's disease DNA methylation among diverse Latinos	6,630	2019-03-15	2024-02-29	1302
USA	California Pacific Med Ctr Res Institute	Study of muscle mobility and aging (somma)	6,138	2018-06-15	2023-05-31	1302

Region	Organization	Project Title	Estimated Average Fund/Year (US$1,000)	Start Date	End Date	Research field (ASJC)
USA	University of California Davis	Vascular inflammation and exosomes as mediators in aging and dementia	2,992	2018-03-15	2023-02-28	1302;1307;1312
USA	Henry Ford Health System	Glymphatic and cognitive impairment of aging and diabetes	2,859	2017-09-15	2022-06-30	1302
USA	The Johns Hopkins University	Mechanisms of synaptic aging mediating cognitive and behavioral symptoms of ad	2,046	2017-05-01	2022-04-30	1302
USA	Stanford University	Regulation of immune cell metabolism in aging and Alzheimer's disease role of the kynurenine pathway	2,043	2017-09-15	2022-06-30	1307;1302
USA	University of Arizona	Thymic and peripheral aspects of t cell aging and rejuvenation	1,987	2017-09-15	2022-05-31	1302
USA	Tulane University of Louisiana	Mentoring research excellence in aging and regenerative medicine	2,136	2012-08-01	2017-05-31	1302;2308
EU	Institut National De La Sante Et De La Recherche Medicale (Inserm)	Heart omics in ageing	20,026	2013-02-01	2019-01-31	2705
EU	Eberhard Karls Universitaet Tuebingen	Integrated European omics research project for diagnosis and therapy in rare neuromuscular and neurodegenerative diseases	19,088	2012-10-01	2017-09-30	1303;1311;1312
EU	Karolinska Institutet	Health and the understanding of metabolism aging and nutrition	18,306	2013-10-01	2018-09-30	1311;2716;1307
EU	Universitätsklinikum Heidelberg	Biomarker research alliance for diagnosing heart disease in the ageing European population	17,205	2013-02-01	2017-01-31	2705
EU	Institut National De La Sante Et De La Recherche Medicale	Well aging and the tanycytic control of health	11,179	2019-03-01	2025-02-28	1312;1314;1300
UK	University of Oxford	UKDP: integrated dementia research environment idea (IDEA)	8,975	2015-04-01	2015-08-14	1300;1100
UK	University of Birmingham	Centre for musculoskeletal ageing research	2,584	2012-08-01	2017-09-30	1314

Region	Organization	Project Title	Estimated Average Fund/Year (US$1,000)	Start Date	End Date	Research field (ASJC)
UK	University of Leicester	Telomere length measurement in UK biobank: advancing understanding of biological ageing and age-related diseases	2,720	2015-04-15	2020-04-14	1300;1000;1100
UK	King's College London	Multiscale analysis of b cell responses in ageing	2,351	2014-05-01	2016-04-30	1300
UK	University of Manchester	Response to mechanical stress in ageing tissue	1,514	2014-09-22	2019-09-21	1307;1312;2737
Japan	Kyoto University	Altered function and structure of immune system in ageing and diseases	1,455	2012-06-28	2017-03-31	1000
Japan	Nagoya University	Brain protein aging and dementia control	1,436	2014-07-10	2019-03-31	1302
Japan	The University of Tokyo	Elucidation of mechanisms of the development of auditory and balance disorders associated with aging and establishment of the treatment strategy against them	389	2014-04-01	2017-03-31	1314;1312;1307
Japan	The University of Tokyo	Elucidation of the molecular mechanisms of aging using mice with extended longevity	376	2017-04-01	2020-03-31	1302;1307
Japan	J. F. Oberlin University	Mechanisms and regulation of socioeconomic disparities in health among the elderly	280	2018-04-01	2023-03-31	2307
Japan	Keio University	Motor and cognitive function measurement system for prevention of falls in the elderly	212	2013-04-01	2016-03-31	3105;2208
Japan	Kyoto University	Developing happy aging community by integrated health data	168	2017-07-18	2020-03-31	1302
Korea	Korea Basic Science Institute	Older animals facilities improve growth and aging research facility environment	1,845	2016-01-01	2017-12-31	1302
Korea	Gwangju Institute of Science and Technology (GIST)	Biological aging characterization study	1,289	2015-01-01	2024-12-31	1302
Korea	Korea Research Institute of Bioscience and Biotechnology	Age-related chronic diseases aging corresponding source control technology	1,463	2012-01-01	2014-12-31	1303;1312
Korea	Gyeongsang National University	Antiaging biomedical science research center	1,038	2015-10-01	2022-02-28	1302;1307;1312

Region	Organization	Project Title	Estimated Average Fund/Year (US$1,000)	Start Date	End Date	Research field (ASJC)
Korea	Korea Research Institute of Bioscience and Biotechnology	Bio-based materials derived from reverse aging effect aging control core technology development	692	2019-01-01	2023-12-31	1302
Korea	Yonsei University	Stem cells derived from specific midbrain dopaminergic neurons of Parkinson's disease using cell therapy research commercialization	576	2012-06-01	2020-05-31	1307;2400
Korea	Korea Research Institute of Bioscience and Biotechnology	Musculoskeletal aging factor discovery and control technology development	1,271	2015-01-01	2018-12-31	1302;1312

※ Research field: 1311- Genetics; 1302-Aging; 1307-Cell Biology; 2705-Cardiology and Cardiovascular Medicine; 1303-Biochemistry; 1311-Genetics; 1312-Molecular Biology; 1300-General Biochemistry, Genetics and Molecular Biology; 1314-Physiology; 1000-Multidisciplinary; 2307-Health, Toxicology and Mutagenesis; 3105-Instrumentation; 2716-Genetics (clinical); 2308-Management, Monitoring, Policy and Law; 1100-General Agricultural and Biological Sciences; 2208-Electrical and Electronic Engineering; 2400-General Immunology and Microbiology; 2737-Physiology (medical)

Table S3. Anti-aging medicine and substances (Cluster 2).

Region	Organization	Project Title	Estimated Average Fund/Year (US$1,000)	Start Date	End Date	Research field (ASJC)
USA	Emory University	Open drug discovery center for Alzheimer's disease(Open-AD)	7,499	2019-09-30	2024-08-31	3002;1313;3004
USA	Indiana Univ-Purdue Univ At Indianapolis	ISUM Alzheimer's disease drug discovery center	5,774	2019-09-30	2024-08-31	1313;3002
USA	University of California San Diego	A Seamless Phase 2A-B Randomized Double Blind Placebo Controlled Trial to Evaluate the Efficacy and Safety of PQ 912 in Patients with Early Alzheimer's Disease	5,279	2019-04-01	2023-06-30	2736
USA	Yale University	Fyn inhibition by azd0530 for Alzheimer's disease	3,794	2013-06-18	2014-07-31	3004
USA	Cognition Therapeutics Inc	Phase 1b first inpatient safety trial for ct1812 a novel Alzheimer's synaptic protection therapeutic	2,411	2016-08-15	2018-07-31	3004;1313;2736
EU	Newron Sweden Ab	Phase II clinical trial of PDGF-BB for the neurological	9,152	2012-01-01	2015-12-31	3004

Region	Organization	Project Title	Estimated Average Fund/Year (US$1,000)	Start Date	End Date	Research field (ASJC)
		regeneration and recovery in Parkinson´s disease				
EU	Universite Du Luxembourg	Systems medicine of mitochondrial Parkinson's disease	6,799	2015-12-01	2019-11-30	1313
EU	Institut National De La Recherche Agronomique	Optimised food products for elderly populations	5,041	2013-09-01	2017-02-28	1106;2916;3601
EU	Biozoon Gmbh	Development of personalised food using rapid manufacturing for the nutrition of elderly consumers	4,561	2012-11-01	2015-10-31	1106
EU	Netherlands Cancer Institute (Stichting Het Nederlands Kanker Instituut-Antoni Van Leeuwenhoek Ziekenhuis)	Senescence therapy for cancer	2,809	2018-10-01	2023-09-30	1313;3004
UK	Newcastle University	Centre for ageing and vitality	3,339	2014-07-01	2019-06-30	2916
UK	Chronos Therapeutics Limited	An integrated nonvertebrate drug discovery platform for neurodegenerative disease	789	2016-03-01	2019-02-28	3004;2736
UK	University College London	Gene therapy for childhood parkinsonism: dopamine transporter deficiency syndrome	652	2018-07-01	2020-12-31	1313
UK	Queen Mary, University of London	The mechanics of the collagen fibrillar network in ageing cartilage	487	2017-10-01	2020-09-30	2204;2502
UK	Cardiff University	Determination of optimal medication to support efficacy of hESC-derived transplants for Parkinson's disease and assessment of side effect risk	272	2017-12-01	2019-11-30	2736
UK	Sentinel Oncology Limited	Validating a new therapeutic opportunity for the treatment of Alzheimer's disease	180	2017-08-01	2018-07-31	3004
UK	Myodopa Limited	Myodopa: Treating Parkinsons diease by transducing constant peripheral secretion of l-dopa	138	2017-12-01	2019-02-28	2736
Japan	The University of Tokyo	Development of a novel antiaging strategy by	1,819	2015-05-29	2020-03-31	1305

Region	Organization	Project Title	Estimated Average Fund/Year (US$1,000)	Start Date	End Date	Research field (ASJC)
		elucidating the mechanisms regulating aging through a muscle centric organ network				
Japan	Japan Women's University	Association between functional alkaline phosphatase gene expression and aging and/or nutritional factors	167	2012-04-01	2016-03-31	2916;2701
Japan	Tokyo Metropolitan Geriatric Hospital and Institute of Gerontology	Effect of vitamin c deficiency on the fetal growth and aging	163	2012-04-01	2015-03-31	2701
Japan	The University of Tokyo	Investigation into functional mechanisms of herbal medicines with attention to sex hormonal effects towards the development of anti-ageing drugs	161	2012-04-01	2015-03-31	3002;2707
Japan	Institute of Physical and Chemical Research	Development of novel glycan-based drug for cancer, COPD and Alzheimer's disease.	159	2015-04-01	2018-03-31	3002;3004
Korea	MEDIPOST Co., Ltd.	Allogeneic cord blood-derived Alzheimer's disease and preterm institutions using mesenchymal stem cell dysplasia drug development	4,230	2012-12-18	2017-12-17	3004;2736
Korea	Dong-A ST	Natural cure Parkinson's disease da9805 us phase completed and foreign technology transfer	2,018	2015-06-01	2019-05-31	2736;3004
Korea	Korea Food Research Institute	Elderly friendly special purpose foods silver foods development	1,601	2014-01-01	2018-12-31	1106;2916;3601
Korea	INIST Corporation	Alzheimer's dementia drug development with immune control proteins	1,778	2017-12-01	2021-12-31	3004;2736;3000
Korea	Korea Research Institute of Bioscience and Biotechnology	Age-related chronic diseases aging corresponding source control technology	1,463	2012-01-01	2014-12-31	3002
Korea	Korea Research Institute of Bioscience and Biotechnology	Agricultural biological micro-biome based material well-aging innovation development and commercialization	1,377	2019-01-01	2023-12-31	2200;2207
Korea	Ildong Pharmaceutical Co.,Ltd	Efficacy and safety study in patients with mild Alzheimer's id1201	1,296	2015-07-20	2018-07-19	3004;2736;1313

※ Research fields: 3002-Drug Discovery; 1313-Molecular Medicine; 2736-Pharmacology (medical); 3004-Pharmacology; 1106-Food Science; 2916-Nutrition and Dietetics; 2204-Biomedical Engineering; 1305-Biotechnology; 2701-Medicine (miscellaneous); 2502-Biomaterials; 2707-Complementary and Alternative Medicine; 3601-Health Professions (miscellaneous); 3000-General Pharmacology, Toxicology and Pharmaceutics

Table S4. Clinical-based research on aging-related diseases, medical services, and policies (Cluster 3)

Region	Organization	Project Title	Estimated Average Fund/Year (US$1,000)	Start Date	End Date	Discipline
USA	Banner Alzheimer's Institute	Alzheimer's prevention initiative	15,253	2012-05-18	2017-04-30	2719;2738;2713
USA	Kaiser Foundation Research Institute	Life course health cerebral pathology and ethnic disparities in dementia	13,079	2016-06-01	2021-05-31	2717;2738
USA	University of California Davis	Epidemiology of age-related dementia, mild cognitive impairment, and brain pathology in a multiethnic cohort of oldest-old	12,173	2017-09-15	2022-06-30	2717;2738
USA	Washington University	Dominantly inherited Alzheimer's network trials unit adaptive prevention trial	5,313	2014-08-01	2020-12-31	2717;2738
USA	University of Southern California	Global Alzheimer's platform trial-ready cohort for preclinical/prodromal Alzheimer's disease	4,985	2017-05-01	2022-04-30	2713;2738;2719
USA	University of Michigan At Ann Arbor	Rehabilitation research and training center (RRTC) on promoting healthy aging for people with long-term physical Disabilities	4,375	2018-09-30	2023-09-29	2717;2909
EU	Universitaet Zuerich	VitaminD3-omega3-home exercise-healthy aging and longevity trial (DO-HEALTH),	14,807	2012-01-01	2017-06-30	2700;2719;2739
EU	The Foundation for Medical Research infrastructural Development And Health Services Next To The Medical Center Tel Aviv	Virtual reality treadmill combined intervention for enhancing mobility and reducing falls in the elderly	8,473	2012-01-01	2015-12-31	2717
EU	Karolinska Institutet	Stress and the aging brain the interplay between genetic susceptibility aging and psychosocial stress on early symptoms of dementia	154	2012-05-01	2013-10-31	2717;2738

Region	Organization	Project Title	Estimated Average Fund/Year (US$1,000)	Start Date	End Date	Discipline
EU	Bar Ilan University	Discovery of drugs for the treatment and prevention of Alzheimer's disease	113	2012-08-01	2016-07-31	2738
EU	Migal Galilee Research Institute Ltd	Centre of excellence for research on environment health and aging	6,151	2012-10-01	2016-03-31	2308
UK	Bangor University	Living well with dementia	5,462	2014-01-01	2015-02-28	2717;2738;2909
UK	University of Manchester	Neighbourhoods and dementia a mixed methods study	5,387	2014-05-01	2019-10-31	2717;2719;2909
UK	University College London	The apple tree programme active prevention in people at risk of dementia through lifestyle behaviour change and technology to build resilience	5,114	2019-01-01	2023-12-31	2717;2738;2719
UK	University of Sussex	Determinants of quality of life, care and costs, and consequences of inequalities in people with dementia and their carers (DETERMIND)	4,986	2019-01-01	2023-12-31	2717;2909;2900
UK	University College London	Empowering better end of life dementia care	4,897	2019-01-01	2023-12-31	2900;2717;2719
Japan	Kyoto University	Novel preventive strategy for Alzheimer's disease based on the toxic conformation theory of amyloid beta	1,496	2014-05-30	2019-03-31	2717
Japan	Kyoto University	Lifestyle and brain function inquiry in psychological science into successful aging	1,204	2016-05-31	2021-03-31	2909;2717
Japan	Kyoto Gakuen University	Formulation and evaluation of physical activity criteria for the prevention of sarcopenia/long-term care applicable to a wide range of elderly	432	2012-04-01	2016-03-31	2739;2900
Japan	National Center For Geriatrics And Gerontology	Development of risk detection system at the early stage of dementia using brain MRI and construction of brain MRI databank in the older adults	414	2019-04-01	2022-03-31	2717;2909;2900
Japan	Saitama University	Techno-sociological research on systems for supporting the daily lives and co-presence of the elderly and migrants with their hometowns	413	2019-04-01	2023-03-31	2717

Region	Organization	Project Title	Estimated Average Fund/Year (US$1,000)	Start Date	End Date	Discipline
Japan	Kobe University	Assisting self care and mutual aid of elderly people with dementia at home based on mind externalization	412	2019-04-01	2023-03-31	2717;2909;2900
Japan	Nagoya Institute of Technology	A cross-sectional study on disease and regional diversity of cognitive impairment in elderly using speech analysis and cerebral blood flow activation	412	2019-04-01	2024-03-31	2717
Japan	Kyoto Institute of Technology	The system for sharing the memory between elderly and care staffs to build a desirable human relationship	394	2015-04-01	2018-03-31	2717;2909
Korea	Korea Basic Science Institute	Older animals facilities improve growth and aging research facility environment	1,845	2016-01-01	2017-12-31	2717;2909
Korea	Korea Institute of Science and Technology	The elderly disabled target daily disability prevention and overcoming technological development	2,117	2019-01-01	2027-04-30	3105
Korea	Ewha Womans University	Development of robot technology for the enhancement of cognitive function and emotional behavior stable mild cognitive impairment and dementia	1,619	2016-05-01	2020-04-30	2738;2719;2717
Korea	Electronics and Telecommunications Research Institute	Noncontact screening of dementia and cognitive sensory function enhancement technology content	1,304	2019-04-01	2021-12-31	2738
Korea	Asan Medical Center	Clinical dementia rating networking	1,080	2012-11-01	2018-10-31	2738

※ Research field: 2719-Health Policy; 2717-Geriatrics and Gerontology; 2713-Epidemiology; 2700-General Medicine; 2738-Psychiatry and Mental Health; 2308-Management, Monitoring, Policy and Law; 2900-General Nursing; 2909-Gerontology; 2739-Public Health, Environmental and Occupational Health; 3105-Instrumentation

Table S5. Aging-related impairment of the brain and cognition (Cluster 4)

Region	Organization	Project Title	Estimated Average Fund/Year (US$1,000)	Start Date	End Date	Research field (ASJC)
USA	Banner Alzheimer's Institute	Alzheimer's prevention initiative apoe4 trial	33,260	2013-09-20	2018-06-30	2728

Region	Organization	Project Title	Estimated Average Fund/Year (US$1,000)	Start Date	End Date	Research field (ASJC)
USA	Kaiser Foundation Research Institute	Life course health cerebral pathology and ethnic disparities in dementia	13,079	2016-06-01	2021-05-31	2800
USA	Mayo Clinic	Prevention of Alzheimer's disease in women: risks and benefits of hormone therapy	10,639	2017-09-15	2022-06-30	2800
USA	University of Southern California	Combination anti-amyloid therapy for preclinical Alzheimer's disease	9,022	2018-09-30	2023-05-31	2800
USA	University of Arizona	Allopregnanolone as regenerative therapeutic for Alzheimer's phase clinical trial	8,411	2019-08-15	2024-04-30	2800;2808
USA	Indiana Univ-Purdue Univ At Indianapolis	Early onset Alzheimer's disease consortium	7,654	2017-09-15	2018-08-31	2800;2808
EU	Stichting VUmc	Amyloid imaging to prevent Alzheimer's disease(AMYPAD)	30,967	2016-10-01	2021-09-30	2800
EU	Westfaelische Wilhelms-Universitaet Muenster	Imaging of neuroinflammation in neurodegenerative diseases	28,224	2012-03-01	2017-02-28	2808;2728
EU	The Chancellor, Masters And Scholars of The University of Oxford	Inhibiting misfolded protein propagation in neurodegenerative diseases	12,876	2017-03-01	2021-02-28	2800;2804
EU	Helsingin Yliopisto	Clinical study in Parkinson's disease with two unique goals: 1) Proof-of-concept of CDNF protein for disease modification; 2) Validation of clinically tested device for intracerebral drug delivery	9,864	2017-01-01	2019-12-31	2728
EU	Institut National De La Sante Et De La Recherche Medicale (Inserm)	Systems biology of pathways involving brain ageing	9,317	2013-01-01	2016-12-31	2800
EU	Newron Sweden Ab	Phase II clinical trial of PDGF-BB for the neurological regeneration and recovery in Parkinson´s disease	9,152	2012-01-01	2015-12-31	2808;2728
UK	University of Oxford	UKDP integrated dementia research environment idea (IDEA)	8,975	2015-04-01	2015-08-14	2800
UK	University of Edinburgh	Centre for cognitive ageing amp cognitive epidemiology	4,014	2013-09-01	2019-08-31	2728

Region	Organization	Project Title	Estimated Average Fund/Year (US$1,000)	Start Date	End Date	Research field (ASJC)
UK	Cardiff University	Further defining the genetic architecture of Alzheimer's disease	3,606	2013-08-29	2019-01-31	2800
UK	University College London	The UK genetic frontotemporal dementia initiative (GENFI)	3,441	2015-05-01	2020-04-30	2800
Japan	Kyoto University	Novel preventive strategy for Alzheimer's disease based on the toxic conformation theory of amyloid beta	1,496	2014-05-30	2019-03-31	2805;2800
Japan	Juntendo University	Development of autophagy enhancing chemicals based on Parkinson's disease pathogenesis	959	2013-06-28	2018-03-31	2804;2728
Japan	Kyushu University	Epidemiological study of stroke, dementia, and depression based on brain MRI in an elderly population: the Hisayama Study Research Project	428	2013-04-01	2016-03-31	2808;2728
Japan	Niigata University	Approach to neurodegenerative disorders by elucidation of the excretion pathway of the brain	414	2019-04-01	2022-03-31	2808;2728
Japan	Juntendo University	Elucidation of the mechanisms of the Parkinson's disease genes and Lewy Body formation and alpha-synuclein propagation mechanisms	407	2018-04-01	2021-03-31	2728;2804
Japan	Kyoto University	Very early diagnosis of Alzheimer's disease by the toxic oligomer specific antibody of amyloid beta	407	2019-04-01	2024-03-31	2805;2800
Japan	Okayama University	Investigation of the brain function network of the cognitive memory and development of an early detection system for dementia	406	2013-05-31	2017-03-31	2728;2808
Korea	Korea Institute of Science and Technology	Older generations predict Alzheimer's early treatments and patient care technology	4,975	2015-12-01	2021-11-30	2728
Korea	Dong-A ST	Natural cure Parkinson's disease da9805 us phase completed and foreign technology transfer	2,018	2015-06-01	2019-05-31	2728
Korea	Korea Institute of Science and Technology	Alzheimer's beta amyloid based diagnostic system development	2,234	2013-04-01	2016-03-31	2800

Region	Organization	Project Title	Estimated Average Fund/Year (US$1,000)	Start Date	End Date	Research field (ASJC)
Korea	Korea Institute of Science and Technology	Older generations predict Alzheimer's early treatments and patient care technology development	1,593	2016-01-01	2021-12-31	2500;2211;2210
Korea	Electronics and Telecommunications Research Institute	Noncontact screening of dementia and cognitive sensory function enhancement technology content	1,304	2019-04-01	2021-12-31	2800
Korea	Asan Medical Center	Clinical dementia rating networking	1,080	2012-11-01	2018-10-31	2728;2800

※ Research field: 2728-Neurology (clinical); 2800-General Neuroscience; 2808-Neurology; 2805-Cognitive Neuroscience; 2804-Cellular and Molecular Neuroscience; 2500-General Materials Science; 2210-Mechanical Engineering; 2211-Mechanics of Materials

Table S6. Smart care for older adults (Cluster 5)

Region	Organization	Project Title	Estimated Average Fund/Year (US$1,000)	Start Date	End Date	Research field (ASJC)
USA	University of Michigan At Ann Arbor	Rehabilitation research and training center (RRTC) on promoting healthy aging for people with long-term physical disabilities	4,375	2018-09-30	2023-09-29	2742
USA	University of Illinois At Urbana-Champaign	Nri:collaborative research aspire automation supporting prolonged independent residence for the elderly	1,296	2015-09-01	2019-08-31	1709;1706;2207
USA	University of Pennsylvania	PFI: BIC Affordable and mobile assistive robots for elderly care	802	2014-08-01	2018-07-31	1700;2207;2200
USA	University of Michigan At Ann Arbor	Factors in aging best practices in archiving and sharing longitudinal data resources on aging	561	2017-09-01	2022-03-31	1706
USA	Zansors Llc	Wireless sensor and telemedicine to screen for sleep apnea in elderly adults	746	2014-09-04	2019-03-31	2200
USA	William D Shannon Consulting Llc	Software Platform for Analyzing Alzheimer's and Parkinson's fMRI Connectomes	552	2016-05-15	2018-08-31	1706
USA	Charles River Analytics Inc	Socially assistive robots for Alzheimer's (SARA)	535	2019-09-15	2021-08-31	1709

Region	Organization	Project Title	Estimated Average Fund/Year (US$1,000)	Start Date	End Date	Research field (ASJC)
EU	Medtronic Iberica Sa	Activating Innovative IoT smart living environments for ageing well	29,203	2017-01-01	2020-06-30	1706;1710;3105
EU	Scuola Superiore Di Studi Universitari E Di Perfezionamento S Anna	Pilots for healthy and active ageing	24,157	2019-12-01	2023-11-30	1710;1706;1700
EU	Charité – Universitätsmedizin Berlin	Personalized recommendations for neurodegenerative disease	17,015	2018-12-01	2022-11-30	1706;1712
EU	Scuola Superiore Di Studi Universitari E Di Perfezionamento Sant'Anna	Implementation and integration of advanced robotic systems and intelligent environments in real scenarios for the ageing population	9,567	2012-01-01	2015-12-31	2606;2207;1702
EU	The Foundation For Medical Researchinfrastructural Development And Health Services Next To The Medical Center Tel Aviv	Virtual reality treadmill combined intervention for enhancing mobility and reducing falls in the elderly	8,473	2012-01-01	2015-12-31	2742;3612
EU	Universita Degli Studi Di Milano	Multiple-actors Virtual Empathic Caregivers for the Elderly	6,723	2017-01-01	2019-12-31	1705;1706;1712
EU	Politecnico Di Torino	Active aGeIng and Osteoporosis: The next challenge for smarT nanobiOmaterials and 3D technologies	6,413	2019-01-01	2023-02-28	2732;2500
EU	Ethniko Kentro Erevnas Kai Technologikis Anaptyxis	Virtual physiological and computational neuromuscular models for the predictive treatment of Parkinson's/nDisease	4,738	2014-01-01	2016-12-31	2611;1712
UK	Heriot-Watt University	Ageing well in urban environments: developing age friendly cities and communities	471	2018-05-15	2020-05-14	2301
UK	Keele University	Recognition of the ageing face	310	2018-06-25	2021-06-24	2216;1702
UK	University of Manchester	Urban ageing and social exclusion	295	2016-03-01	2020-02-29	2105;2301
UK	Gingersnap Studios Limited	Elderberry platform prototype	284	2014-02-01	2015-01-31	1706;1709
UK	Dolphin Computer Access Limited	Ables accessibility brought to low vision and elderly sectors	130	2013-08-01	2014-01-31	1705;1706;2208

Region	Organization	Project Title	Estimated Average Fund/Year (US$1,000)	Start Date	End Date	Research field (ASJC)
UK	University of Brighton	Electrochemical sensor for monitoring levels of oxygen and nitrogen reactive species to benefit ageing research	124	2013-09-23	2014-09-22	3105
Japan	The University of Tokushima	Development of the method of nursing as caring for the elderly by collaborating with humanoid interactive robot	382	2017-04-01	2022-03-31	1709
Japan	Kyushu University	A study of game design through the development of locomotive syndrome measures game for elderly	370	2013-04-01	2016-03-31	3612;2732
Japan	Keio University	Motor and cognitive function measurement system for prevention of falls in the elderly	212	2013-04-01	2016-03-31	3105;2208;1706
Japan	Keio University	Living space supporting solitary elderly who lives by oneself using supporting robot	156	2015-04-01	2018-03-31	1709;2207;1706
Japan	Shinshu University	Application of robotic wear curara for intractable neurodegenerative diseases	151	2015-04-01	2018-03-31	2742
Korea	Pusan National University	Infrastructure and product development for the antiaging industry support	5,719	2012-12-01	2015-11-30	2216;1700;2200
Korea	Korea Institute of Science and Technology	Connected active space cas technology development projects for the elderly life support	4,188	2015-01-01	2023-12-31	1709;1706;1705
Korea	Electronics and Telecommunications Research Institute	Room environment human care robotic technology to cope with aging society	5,022	2017-04-01	2021-12-31	1709;1706;2208
Korea	Korea Institute of Construction Technology	Slope surveyed and inspected improving and reinforcing the aging facility corresponding technology	2,009	2019-04-08	2021-12-31	2205;1909;2208
Korea	Soongsil University	Big databased service robot developed to provide an aging generation of individual specific social services	994	2014-07-01	2018-06-30	1705;1700;1708
Korea	Neo Crema Ltd	Elderly-friendly customized functional materials developed by local agricultural resources	1,048	2015-05-01	2018-04-30	2200
Korea	Korea Electronics Technology Institute	Web objects web objects-based self paced smart aging ageing service development	1,068	2015-09-01	2018-08-31	1710;1700;3105

※ Research field: 2742-Rehabilitation; 1709-Human-Computer Interaction; 1700-General Computer Science; 1706-Computer Science Applications; 2200-General Engineering; 1710-Information Systems; 2606-Control and Optimization; 1705-Computer Networks and Communications; 2732-Orthopedics and Sports Medicine; 2611-Modeling and Simulation; 2301-Environmental Science (miscellaneous); 2216-Architecture; 2105-Renewable Energy, Sustainability and the Environment; 3105-Instrumentation; 3612-Physical Therapy, Sports Therapy and Rehabilitation; 2205-Civil and Structural Engineering; 2207-Control and Systems Engineering; 1712-Software; 2500-General Materials Science; 1702-Artificial Intelligence; 2208-Electrical and Electronic Engineering; 1909-Geotechnical Engineering and Engineering Geology; 1708-Hardware and Architecture

Table S7. Aging of the immune system (Cluster 6):

Region	Organization	Project Title	Estimated Average Fund/Year (US$1,000)	Start Date	End Date	Research field (ASJC)
USA	The Regents of University of California	Establishment to senescence: plant-microbe and microbe-microbe interactions mediate switchgrass sustainability	7,375	2015-08-15	2020-08-14	2303
USA	Mount Sinai School of Medicine	Systematic drug repurposing targeting immune activation networks in Alzheimer's disease	4,186	2018-07-15	2023-06-30	2403
USA	Baylor College of Medicine	Interrogating the interferon pathway in aging and Alzheimer's disease	2,881	2018-06-01	2023-05-31	2403
USA	Agricultural Research Service	Immunity inflammation and nutrition in aging	1,528	2014-10-01	2019-09-30	2403
USA	Stanford University	Effects of aging on primary and secondary vaccine responses in a 15year longitudinal cohort	873	2017-04-01	2022-03-31	2403;2723
EU	Universitair Medisch Centrum Utrecht	Vaccines and infectious diseases in the ageing population	14,081	2019-01-01	2023-12-31	2403;2725
EU	Consiglio Nazionale Delle Ricerche	An integrated approach to dissect determinants risk factors and pathways of ageing of the immune system	6,629	2015-05-01	2019-04-30	2403
EU	The Babraham Institute	ToWards Immunisations that Last: the Immunology and Gerontology of Helper T cells	1,700	2015-06-01	2020-05-31	2403;2723
EU	Ecole Polytechnique Federale De Lausanne	Exploring the link between innate immunity and cellular aging	1,688	2019-01-01	2023-12-31	2403
EU	Kobenhavns Universitet	Age-associated signatures in the composition and proinflammatory status of the gut microbiome in humans and mice and the impact of a periodic fasting intervention to promote healthy aging	227	2016-03-01	2018-02-28	2404

Region	Organization	Project Title	Estimated Average Fund/Year (US$1,000)	Start Date	End Date	Research field (ASJC)
EU	Centre National De La Recherche Scientifique Cnrs	Control of central nervous system inflammation by meningeal macrophages and its impairment upon aging	209	2019-05-01	2021-04-30	2403
UK	King's College London	Multi-scale analysis of B cell responses in ageing (MABRA)	2,351	2014-05-01	2016-04-30	2403;2723
UK	University College London	The integration of human t cell senescence and function at the molecular level	768	2016-10-10	2020-04-15	2403
UK	Liverpool School of Tropical Medicine	Epidemiological consequences of reproductive senescence in a long-lived vector	509	2017-09-01	2020-08-31	1109;2405;2725
UK	Babraham Institute	Immunity in health and ageing dissecting the role of the noncanonical autophagy pathway	480	2018-11-05	2021-11-04	2403;2723
UK	Royal Veterinary College	Immunosenescence in geriatric dogs and impact on vaccination	132	2012-10-01	2016-09-30	3400
Japan	Kyoto University	Altered function and structure of immune system in ageing and diseases	1,455	2012-06-28	2017-03-31	2403;2723
Japan	Juntendo University	Development of autophagy enhancing chemicals based on Parkinson's disease pathogenesis	959	2013-06-28	2018-03-31	2403
Japan	Niigata University	Signalling Mechanism of Autophagy Regulation by Nutrients and Food Components : Its Diversity and Anti-aging	153	2014-04-01	2017-03-31	2404
Japan	Keio University	Elucidation of molecular basis common to cardiovascular disorders associated with aging and metabolic syndrome	161	2015-04-01	2018-03-31	2403
Japan	Osaka University	Epidemiological analyses for the impact of bacterial and virus infection to the occurrence of dementia by long-term cohort studies	400	2018-04-01	2022-03-31	2725;2404
Korea	Korea Research Institute of Bioscience and Biotechnology	Aging immune modulators excavation and immunotherapy technology development	1,053	2015-01-01	2018-12-31	2403;2723
Korea	Genechem Co., Ltd.	Global functional materials development and commercialization of immune enhancing material sialyl lactose	647	2018-07-01	2020-12-31	2745

Region	Organization	Project Title	Estimated Average Fund/Year (US$1,000)	Start Date	End Date	Research field (ASJC)
		to improve the quality of life for an aging society				
Korea	Yonsei University	Aging cohort of inflammatory immune mechanism and researchers	338	2019-09-01	2022-02-28	2723;2403
Korea	Korea University	Geriatric treatment target excavation of severe respiratory inflammation and control research	270	2017-06-30	2022-06-29	2740;2403;2723
Korea	Heath Balance Co., Ltd.	Grain formula development and commercialization for the elderly nutrition and immune enhancement	177	2014-11-28	2016-11-27	2209;2200

※ Research field: 2303-Ecology; 2403-Immunology; 2404-Microbiology; 1109-Insect Science; 3400-General Veterinary; 2725-Infectious Diseases; 2745-Rheumatology; 2723-Immunology and Allergy; 2740-Pulmonary and Respiratory Medicine; 2209-Industrial and Manufacturing Engineering; 2405-Parasitology; 2200-General Engineering;
